# Cost Analysis of Abdominal Aortic Aneurysm Repair: The Economic Impact of Rising Surgical Material Costs on Public Health Insurance

**DOI:** 10.3400/avd.oa.25-00109

**Published:** 2026-02-07

**Authors:** Etsuji Umeda, Kiyoshi Doi, Osamu Sakai, Takayoshi Kato, Hiroki Ogura, Masayuki Sato

**Affiliations:** Department of Cardiovascular Surgery, Graduate School of Medicine, Gifu University, Gifu, Gifu, Japan

**Keywords:** abdominal aortic aneurysm, endovascular aneurysm repair, open aneurysm repair, hospitalization costs, propensity score matching

## Abstract

**Objectives:**

We conducted a detailed comparison of inpatient medical costs between endovascular aneurysm repair (EVAR) and open aneurysm repair (OAR) for abdominal aortic aneurysms.

**Methods:**

We evaluated 312 EVAR and 205 OAR cases performed at our institution between January 2007 and December 2017. Patient background characteristics were adjusted between the EVAR and OAR groups using propensity score matching (PSM). Surgical outcomes and inpatient medical costs were compared.

**Results:**

After PSM, 161 cases were included in each group for comparison. Operative time, blood loss, intensive care unit (ICU) stay, and hospital stay were significantly lower in the EVAR group than in the OAR group. Total inpatient medical costs were significantly higher in the EVAR group (3111× 10^3^ vs. 2156 × 10^3^ JPY [Japanese yen], p <0.01). The surgical material costs in the EVAR group were significantly higher than those in the OAR group, accounting for 58% of total medical expenses. Other costs (diagnosis procedure combination, ICU management, surgical procedure, transfusion, intraoperative injection, and room) were all lower in the EVAR group than in the OAR group.

**Conclusions:**

The cost-saving effects of EVAR, such as reduced transfusion costs and ICU stay fees, were offset by the significantly higher cost of surgical materials.

## Introduction

Abdominal aortic aneurysms (AAAs) are treated using 2 primary approaches: open aneurysm repair (OAR) and endovascular aneurysm repair (EVAR). Although EVAR is associated with significantly higher costs due to the use of stent grafts, it offers potential cost savings by reducing blood transfusion requirements and shortening hospital stays. Thus, numerous studies have compared the hospitalization costs associated with OAR and EVAR.

Three previous randomized prospective studies conducted on low-risk patients have reported conflicting findings; 2 studies indicate that EVAR incurred higher hospitalization costs than did OAR.^1–3)^ However, these studies do not fully reflect real-world clinical settings, particularly those including high-risk patients. Additionally, retrospective studies comparing hospitalization costs have yielded inconsistent conclusions, often excluding emergency cases or lacking proper adjustments for differences in patient backgrounds, limiting the reliability of their findings.^4–17)^

To the best of our knowledge, this study is the first to comprehensively compare hospitalization costs between EVAR and OAR, which included all cases (emergency and elective) managed at a single institution. Applying propensity score matching (PSM) and inverse probability treatment weighting (IPTW) to adjust for differences in patient backgrounds, we offer a more precise and clinically relevant cost analysis.

## Materials and Methods

### Patient selection

This study evaluated 517 cases of AAA surgery, including emergency cases, performed at our institution between January 1, 2007 and December 31, 2017. At our institution, EVAR is the first-choice treatment in patients aged ≥75 years, provided that their anatomy is suitable. As a result, 205 and 312 patients underwent OAR and EVAR, respectively. These 2 groups were compared.

### Breakdown of hospitalization costs

Japan's healthcare system operates under the Diagnosis Procedure Combination (DPC) system.^18)^ Hospitalization costs comprised a fixed-payment part of the DPC-based payment system and a fee-for-service part as follows: The fixed-payment DPC part included hospitalization costs, oral medication costs, injectable drugs administered outside the operating room, procedural fees, diagnostic test fees, pathological examination fees, and imaging fees. The fee-for-service part included surgical procedure fees, anesthesia management fees, intensive care unit (ICU) management fees, surgical material costs, blood product costs, intraoperative injectable drug costs, meal charges, and room charges. Since the study included Japanese cases under the Japanese DPC system, the results are reported in Japanese yen (JPY). However, for international readers the total cost is converted into euros and also reported. The exchange rate between the euro and the JPY was calculated (1 euro = 123 yen, as of December 13, 2021).

### Patient background factors used for analysis

The following patient characteristics were analyzed: age, gender, body mass index (BMI), aneurysm diameter, hemoglobin (Hb) level, history of coronary artery disease, cerebral infarction, diabetes mellitus (HbA1c >6.5%), heart failure (ejection fraction <40%), dyslipidemia and hypertension (based on medication use), chronic obstructive pulmonary disease (COPD) (based on inhaled medication use), chronic kidney disease (CKD) (estimated glomerular filtration rate <60 mL/min/1.73 m^2^), atrial fibrillation, malignancy, hypertension, history of laparotomy, and ruptured aneurysm cases.

### Study outcomes and statistical analysis

Four key aspects were investigated: (1) factors that influenced total inpatient costs, and a multivariate analysis (linear regression) was used for assessing contributing factors; (2) comparison of surgical outcomes (operative time, blood loss, ICU stay, postoperative hospital stay, and mortality) between EVAR and OAR; (3) comparison of total inpatient medical costs between EVAR and OAR; and (4) comparison of the breakdown of inpatient medical costs between EVAR and OAR.

Regarding analyses of aspects 2–4, PSM and IPTW were used for adjusting for confounding variables. Patient characteristics are summarized as median and interquartile range (IQR) for continuous variables and as frequencies for categorical variables. A multivariable linear regression analysis was performed to identify the factors that strongly affected total inpatient costs. Since the distribution of total hospitalization costs was skewed, a natural logarithmic transformation was applied; moreover, the regression coefficients were presented following an inverse transformation.

We performed an analysis using the propensity scores to compare operative time, bleeding, ICU stay, postoperative length of hospital stay, and 30-day mortality between EVAR and OAR, while controlling for confounding factors. A logistic regression model was used for estimating the propensity scores. The following variables were included as covariates: age, gender, BMI, AAA diameter, Hb level, coronary artery disease, cerebral infarction, diabetes mellitus, heart failure, dyslipidemia, COPD, CKD, atrial fibrillation, malignant tumor, hypertension, post-abdominal operation, and rupture. In the 1:1 PSM, the caliper was set to a standard deviation of 0.25 of the linear predicted value obtained from the logistic regression model. As a sensitivity analysis, an IPTW analysis was also performed. Total hospitalization costs and cost components for EVAR and OAR are summarized using the median and IQR, and were compared using the Mann–Whitney U test. The 2-sided significance level was set at 5%. A 2-sided p <0.05 was considered statistically significant. All analyses were performed using R version 4.4.1 (The R Project for Statistical Computing, Vienna, Austria; https://www.r-project.org).

## Results

Among the 517 patients, the median age was 75 (69–80) years, and 457 (88%) were male participants. The median maximum short-axis diameter of AAA was 52 (47–57) mm, and the median Hb level was 13.1 (11.5–14.2) g/dL. Background diseases included coronary artery disease in 158 (31%) patients, cerebral infarction in 68 (13%), diabetes in 107 (21%), heart failure in 7 (1%), dyslipidemia in 241 (47%), COPD in 39 (8%), CKD in 228 (44%), atrial fibrillation in 39 (8%), malignancy in 110 (21%), hypertension in 405 (78%), history of laparotomy in 114 (22%), and ruptured aneurysms in 39 (8%) (**Table 1**).

**Table 1:** Patient background characteristics

Number of cases	517
Age (year)	75 (69–80)
Male gender	457 (88%)
BMI (kg/m^2^)	23.1 (21–24.8)
AAA diameter (mm)	52 (47–57)
Hb (g/dL)	13.1 (11.5–14.2)
Coronary artery disease	158 (31%)
Cerebral infarction	68 (13%)
Diabetes mellitus	107 (21%)
Heart failure	7 (1%)
Dyslipidemia	241 (47%)
COPD	39 (8%)
Chronic kidney disease	228 (44%)
Atrial fibrillation	39 (8%)
Malignant tumor	110 (21%)
Hypertension	405 (78%)
History of laparotomy	114 (22%)
AAA rupture	39 (8%)
EVAR	312 (60%)

Categorical variables are presented as number (%). Continuous variables are presented as medians (interquartile range).

AAA: abdominal aortic aneurysm; BMI: body mass index; COPD: chronic obstructive pulmonary disease; EVAR: endovascular aneurysm repair; Hb: hemoglobin

### Factors influencing total hospitalization costs

Multivariate analysis identified the following independent factors that significantly increased total hospitalization costs: EVAR, AAA diameter, anemia, atrial fibrillation, and AAA rupture (**Table 2**).

**Table 2:** Factors influencing total hospitalization costs

Variables	Exp (β)	95% LCI	95% UCI	p-Value
Age (year)	1.00	1.00	1.01	0.13
Male gender	1.00	0.93	1.08	0.99
BMI (kg/m^2^)	0.99	0.96	1.02	0.59
AAA diameter (mm)	1.00	1.00	1.00	0.02
Hb (g/dL)	0.94	0.90	0.97	<0.01
Coronary artery disease	1.04	0.99	1.10	0.11
Cerebral infarction	1.01	0.95	1.08	0.73
Diabetes mellitus	0.99	0.94	1.05	0.74
Heart failure	0.84	0.69	1.03	0.09
Dyslipidemia	1.03	0.98	1.08	0.21
COPD	1.00	0.91	1.09	0.94
Chronic kidney disease	1.05	1.00	1.10	0.08
Atrial fibrillation	1.18	1.08	1.28	<0.01
Malignant tumor	1.05	0.99	1.11	0.08
Hypertension	1.02	0.97	1.08	0.41
History of laparotomy	1.01	0.96	1.07	0.69
AAA rupture	1.68	1.53	1.86	<0.01
EVAR	1.46	1.39	1.54	<0.01

AAA: abdominal aortic aneurysm; BMI: body mass index; COPD: chronic obstructive pulmonary disease; EVAR: endovascular aneurysm repair; LCI: lower limit of confidence interval; UCI: upper limit of confidence interval; Hb: hemoglobin

The EVAR group had significantly older age and malignancy rates than did the OAR group, whereas the OAR group had significantly larger aneurysm sizes and more rupture cases than did the EVAR group (**Table 3**).

**Table 3:** Comparison of patient characteristics between EVAR and OAR

	EVAR	OAR	p-Value
Number of cases	312	205	
Age (year)	77 (71–82)	71 (65–77)	<0.01
Male gender (%)	277 (88.8%)	181 (88.3%)	0.97
BMI (kg/m^2^)	23.0 (20.9–24.7)	23.2 (21.2–25.0)	0.57
AAA diameter (mm)	51 (47–56)	53 (48–61)	<0.01
Hb (g/dL)	13.0 (11.4–14.1)	13.2 (11.8–14.3)	0.28
Coronary artery disease	94 (30%)	64 (31%)	0.79
Cerebral infarction	43 (14%)	25 (12%)	0.60
Diabetes mellitus	69 (22%)	38 (19%)	0.32
Heart failure	6 (2%)	1 (0.5%)	0.16
Dyslipidemia	137 (44%)	104 (51%)	0.12
COPD	29 (9%)	10 (5%)	0.06
Chronic kidney disease	146 (47%)	82 (40%)	0.14
Atrial fibrillation	23 (7%)	16 (8%)	0.85
Malignant tumor	76 (24%)	34 (17%)	0.04
Hypertension	245 (79%)	160 (78%)	0.89
History of laparotomy	74 (24%)	40 (20%)	0.26
Rupture	14 (4%)	25 (12%)	<0.01

Categorical variables are presented as number (%). Continuous variables are presented as medians (interquartile range).

AAA: abdominal aortic aneurysm; BMI: body mass index; COPD: chronic obstructive pulmonary disease; EVAR: endovascular aneurysm repair; Hb: hemoglobin; OAR: open aneurysm repair

### Surgical outcomes: EVAR vs. OAR

Before adjustment, the EVAR group had significantly shorter operative time, less blood loss, shorter ICU stays, and shorter postoperative hospital stays than did the OAR group (**Table 4**). However, 30-day postoperative mortality did not differ between the groups. This trend remained the same after adjusting for background factors. Details on the surgical technique have been compiled in the Supplementary Data (**Supplementary Table 1**). Since the incidence of postoperative complications might affect treatment costs, supplementary data are presented in **Supplementary Table 2**.

**Table 4:** Comparison of surgical outcomes between EVAR and OAR

	Unadjusted			PSM			IPTW		
	EVAR	OAR	p-Value	EVAR	OAR	p-Value	EVAR	OAR	p-Value
Number of cases	312	205		161	161		518.68	524.79	
Operation time (min)	116 (98–151)	230 (189–277)	<0.01	108 (94–134)	231 (190–276)	<0.01	116 (98–151)	232 (189–279)	<0.01
Bleeding (mL)	80 (40–150)	1344 (890–2100)	<0.01	70 (40–130)	1320 (890–2061)	<0.01	77 (40–151)	1312 (872–2060)	<0.01
ICU stay (days)	1.0 (0.0–1.0)	1.0 (1.0–1.0)	<0.01	1.0 (0.0–1.0)	1.0 (1.0–1.0)	<0.01	1.0 (0.0–1.0)	1.0 (1.0–1.0)	<0.01
Postoperative length of hospital stay (days)	8.0 (7.0–10.0)	13 (11.0–18.0)	<0.01	8.0 (7.0–9.0)	14.0 (11.0–18.0)	<0.01	8 (7, 10)	14 (11–18)	<0.01
30-day mortality (%)	3 (1.0%)	2 (1.0%)	1.0	2 (1.2%)	1 (0.6%)	1.0	9.8 (1.8%)	2.5 (0.5%)	0.11

Continuous variables are presented as medians (interquartile range).

EVAR: endovascular aneurysm repair; ICU: intensive care unit; IPTW: inverse probability treatment weighting; OAR: open aneurysm repair; PSM: propensity score matching

### Total hospitalization costs: EVAR vs. OAR

After PSM adjustment, total hospitalization costs were significantly higher in the EVAR group, at approximately 1.4 times that of the OAR group (3111 × 10^3^ vs. 2156 × 10^3^ JPY, p <0.01) (**Table 5**). The IPTW analysis confirmed this finding.

**Table 5:** Comparison of total hospitalization costs and cost components between EVAR and OAR

	Unadjusted			PSM			IPTW		
	EVAR	OAR	p-Value	EVAR	OAR	p-Value	EVAR	OAR	p-Value
Number of cases	312	205		161	161		518.68	524.79	
Inclusive part									
DPC charges	523 (465–625) 16%	775 (655–937) 36%	<0.01	510 (447–568) 16%	775 (664–945) 36%	<0.01	523 (465–631)	755 (667–915)	<0.01
Fee-for-service part									
Surgical procedure fees	494 (474–494) 15%	543 (543–591) 25%	<0.01	494 (474–494) 16%	543 (520–591) 25%	<0.01	494 (474–494)	543 (520–591)	<0.01
Surgical material costs	2002 (1760–2387) 60%	279 (237–323) 13%	<0.01	1808 (1679–2060) 58%	279 (237–325) 13%	<0.01	2002 (1760–2396)	279 (236–325)	<0.01
Intravenous drug costs	39 (30–52) 1%	42 (32–53) 2%	0.06	35 (29–48) 1%	41 (32–50) 2%	0.01	39 (31–53)	40 (32–50)	0.64
Blood product costs	0 (0–0) 0%	121 (85–219) 6%	<0.01	0 (0–0) 0%	115 (79–212) 5%	<0.01	0 (0–0)	121 (83217)	<0.01
Anesthesia management fees	88 (82–100) 3%	140 (124–166) 6%	<0.01	88 (82–94) 3%	139 (124–165) 6%	<0.01	88 (82–104)	139 (124–168)	<0.01
ICU management fees	72 (0–72) 2%	72 (71–122) 3%	<0.01	72 (0–72) 2%	72 (71–116) 3%	<0.01	72 (0–73)	72 (71–116)	0.01
Meal charges	20 (17–26) 1%	28 (21–35) 1%	<0.01	18 (16–22) 1%	28 (22–37) 1%	<0.01	20 (17–26)	28 (22–37)	<0.01
Hospital room charges	6 (2–15) 0%	9 (3–39) 0%	<0.01	6 (2–12) 0%	9 (3–27) 0%	0.03	6 (3–15)	9 (3–27)	0.09
Total hospitalization costs	3356 (3035–3781)	2157 (1960–2545)	<0.01	3111 (2904–3436)	2156 (1949–2545)	<0.01	3395 (3032– 3885)	2155 (1943–2555)	<0.01
Total hospitalization costs (€)*	27285	17534		25296	17531		27599	17524	

Costs are listed in Japanese yen × 10^3^.

^*^ For international readers, the total hospitalization costs converted to euros are also listed at the bottom of the list (The exchange rate between the euro and the Japanese yen was calculated 1 euro = 123 yen, as of December 13, 2021). Continuous variables are presented as medians (interquartile range). The percentages shown represent the ratio of each expense category's cost to the total hospitalization cost.

DPC: diagnosis procedure combination; EVAR: endovascular aneurysm repair; ICU: intensive care unit; IPTW: inverse probability treatment weighting; OAR: open aneurysm repair; PSM: propensity score matching

### Breakdown of medical costs: EVAR vs. OAR

After PSM adjustment, the EVAR group exhibited significantly higher surgical materials costs than did the OAR group (1808 × 10^3^ vs. 279 × 10^3^ JPY, p <0.01) (**Table 5**). However, all other costs—including DPC charges, ICU management fees, surgical procedure fees, intraoperative injection costs, blood product costs, anesthesia management fees, meal charges, and room charges—were lower in the EVAR group. In the EVAR group, surgical material costs (1808 × 10 JPY) accounted for 58% of the total hospitalization costs (3111 × 10^3^ JPY) (refer to the row for surgical material costs in the column for the EVAR group after PSM in **Table 5**), representing the largest expense item. In the OAR group, the largest expense item was DPC charges (775 × 10^3^ JPY), comprising 36% of the total hospitalization costs (2156 × 10^3^ JPY), followed by surgical procedure fees (543 × 10^3^ JPY, 25%), and the third item was surgical material costs (279 × 10^3^ JPY, 13%). Similar trends were observed when adjusting or background factors using IPTW, with surgical material costs being significantly higher in the EVAR group, whereas all other costs were lower than those in the OAR group.

## Discussion

Japan’s medical expenses amount to 3.3 × 10^14^ JPY, whereas the national budget is 8.1 × 10^14^ JPY, indicating that approximately 40% of the national budget was allocated to healthcare. With Japan's rapidly aging society, controlling these costs has become a pressing issue. According to the 2022 data from the Organization for Economic Co-operation and Development (OECD), the proportion of medical expenses relative to the gross domestic product (GDP) is increasing globally, prompting all countries to implement cost-reduction measures. Japan's medical expenses account for 11.5% of its GDP, ranking as the fourth highest among OECD countries.^19)^ Additionally, Japan has the second-highest aging rate after the Republic of Korea, making cost reduction an urgent national priority.^20)^

In response to efforts to curb medical expenditures, comparisons between 2 major treatments of AAA, OAR, and EVAR, were conducted. Previous retrospective studies on hospitalization costs have yielded inconsistent results, with some indicating higher costs for OAR,^4–8)^ others for EVAR,^9–15)^ and some reporting no significant difference.^16,17)^ These inconsistencies largely stem from inadequate adjustment for differences in patient backgrounds. Among 3 prospective randomized trials, EVAR was found to be more expensive in the EVAR 2005 and DREAM 2007 trials, whereas OAR was more costly in the OVER 2012 trial.^1–3)^ However, these studies only focused on low-risk patients, limiting their applicability to high-risk cases, including emergencies.

To address these limitations, in our study, we performed multivariate analysis to identify factors influencing total hospitalization costs, encompassing both low- and high-risk patients. The results demonstrated that EVAR was an independent risk factor for increased costs, along with aneurysm diameter, anemia, atrial fibrillation, and AAA rupture cases. After adjusting for patient backgrounds using PSM, total hospitalization costs for EVAR were found to be 1.4 times higher than those for OAR (**Table 5**).

A major contributor to the increased cost of EVAR was surgical materials, including aortic stent grafts and artificial blood vessels, which accounted for 58% of total expenses (refer to the row for surgical material costs in the column for EVAR group after PSM in **Table 5**). Other cost components—such as DPC charges, ICU management fees, surgical procedure fees, anesthesia management fees, intravenous drug costs, blood product costs, meal charges, and hospital room charges—were lower in the EVAR group than that in the OAR group. Despite procedural advantages such as shorter operation time, reduced blood loss, and shorter ICU and hospital stays, the high cost of surgical materials outweighed these savings, making EVAR the more expensive option overall. (**Tables 4** and **5**). Rupture cases had an even stronger impact on total costs than did EVAR (**Table 2**). Although background factors could not be standardized due to the small number of cases, even in the sub-analysis of rupture cases alone, surgical material costs accounted for the largest proportion in the EVAR group, which still tended to have higher costs compared with the OAR group (**Supplementary Table 3**).

Material costs fluctuate depending on the country and time period, and total hospitalization costs are influenced by the study population and healthcare system. Nevertheless, our findings align with previous reports, suggesting global relevance. The median surgical material costs for EVAR in our study (1808 × 10^3^ JPY) were slightly higher than in previous studies, despite showing no significant difference.^3,5,11,12,14,16)^ Likewise, the median total hospitalization costs (3111 × 10^3^ JPY) and the proportion of surgical material costs (58%; **Table 5**) were consistent with those in prior research.

Beyond hospitalization costs, EVAR also presents long-term financial considerations. Approximately 20% of EVAR cases experience complications such as endoleak and stent graft migration.^21,22)^ As a result, long-term medical costs, including follow-up imaging and retreatment, tend to be higher for EVAR than for OAR.^2,23)^

The advantage of EVAR over OAR is its minimal invasiveness. Even after adjusting for background factors, EVAR exhibited shorter operative times, ICU stays, and hospital stays, along with reduced blood loss (**Table 4**). In our study, which included high-risk cases such as ruptured aneurysms, no significant difference was observed in in-hospital mortality between EVAR and OAR after PSM adjustment. However, in a previous randomized prospective comparative study of low-risk patients, the 30-day mortality rate of EVAR was significantly lower.^24)^ Additionally, in a study focusing solely on rupture cases with PSM adjustment, the in-hospital mortality rate of EVAR was significantly lower than that of OAR.^25)^ Despite these short-term benefits, long-term studies have reported a higher risk of aneurysm rupture for EVAR compared with OAR during follow-up periods of 3–8 years.^26,27)^ Consequently, EVAR seems best suited for high-risk patients whose short-term outcomes are a priority, whereas OAR may be preferable in patients with longer life expectancy.

In Japan, AAA surgeries are fully covered by public health insurance without a cost ceiling, allowing patient preference to influence treatment selection. In clinical practice, EVAR is often chosen for managing older and high-risk patients due to its minimally invasive nature, despite being more expensive. However, as Japan seeks to enhance cost-effectiveness and reduce healthcare expenditures, this trend contradicts national financial goals. According to the Japanese Society for Vascular Surgery, the proportion of EVAR procedures increased from 52% in 2012 to 63% in 2017.^28,29)^

To ensure the sustainability of Japan’s healthcare system, reducing the cost of surgical materials is imperative. Without such measures, the financial burden on public medical insurance could become unsustainable, potentially limiting access to necessary treatments in older and high-risk patients.

### Limitations of this study

This study has a few limitations. It was conducted at a single institution and involved a relatively small sample size. It is a reasonable inference that aortic aneurysm size influences the incidence of rupture and that ruptured cases affect costs. However, there is 1 previous report indicating that the aneurysm size itself directly impacted costs.^30)^ Statistically, in such cases, “rupture” should be considered as a mediator (intermediate factor). Analyzing both factors (aneurysm size and rupture) simultaneously using multivariate analysis might overestimate the effect of aortic aneurysm size (**Table 2**). Additionally, healthcare systems and medical resource costs vary across countries, which should be considered when interpreting these findings. Future research incorporating broader datasets and international comparisons would enhance the generalizability of these results.

## Conclusion

Hospitalization costs for AAA treatment were significantly higher for EVAR than for OAR, primarily due to the high cost of surgical materials. However, EVAR demonstrated cost-saving advantages in areas such as blood products and ICU stays. To ensure the long-term viability of Japan’s healthcare system, reducing the cost of surgical materials is a crucial step toward managing national medical expenditures.

## Supplementary Information

Supplementary Table 1 Surgery details.

Supplementary Table 2 Postoperative complications.

Supplementary Table 3 Cost comparison between EVAR and OAR in AAA ruptured cases.
