# Supplementary material for: Cost Analysis of Abdominal Aortic Aneurysm Repair: The Economic Impact of Rising Surgical Material Costs on Public Health Insurance
Source: Ann Vasc Dis. 2026 Feb 7;19(1):25-00109. doi: 10.3400/avd.oa.25-00109 (PMC12895106; doi:10.3400/avd.oa.25-00109)
Supplement: Supplementary Table 1 — Surgery details. [file avd-19-1-25-00109-s001.pdf]

Supplementary Table 1 Surgery details

**Endovascular aneurysm repair**	Number of cases
**Endograft type**	
Zenith (Cook Medical, Bloomington, IN, USA)	47
Endurant (Medtronic, Santa Rosa, CA, USA)	81
Excluder (W.L.Gore, Flagstaff, AZ, USA)	168
AFX (Endologix, Irvine, CA, USA)	7
Powerlink (Endologix, Irvine, CA, USA)	9
**Number of graft used**	
1	4
2	101
3	151
4	40
≥5	16
**Concomitant procedure**	
Embolization of the internal iliac artery	105
External-internal iliac artery bypass	10
Femoro-femoral bypass	1
**Open aneurysm repair**	
**Graft type**	
Straight	36
Bifurcated	169
**Concomitant procedure**	
Femoro-popliteal bypass	2

Supplementary Table 2 Postoperative complications

	Unadjusted					PSM				
	EVAR	OAR	Odds ratio	95%CI	P-value	EVAR	OAR	Odds ratio	95%CI	P-value
Number of cases	312	205				161	161			
Ischemic colitis	0	5 (2.4%)			.01	0	4 (2.5%)			.06
Wound infection	4 (1.3%)	15 (7.3%)	.17	.05 - .50	<.01	1 (0.6%)	15 (9.3%)	.06	.01 - .46	<.01
Newly introduced hemodialysis	2 (0.6%)	6 (2.9%)	.21	.04 - 1.07	.06	0	4 (2.5%)			.06
Stroke	0	0				0	0			
Myocardial infarction	0	1 (0.5%)			.40	0	1 (0.6%)			.50
New atrial fibrillation	4 (1.3%)	2 (1.0%)	1.32	.24 - 7.26	1.0	2 (1.2%)	2 (1.2%)			1.0
Long intubation (≧48 hours)	5 (1.6%)	12 (5.9%)	.26	.09 - .76	<.01	3 (1.9%)	10 (6.2%)	.28	.08 - 1.05	.05
Endoleaks	100 (32%)					49 (30%)				
Type 1a	5					2				
Type 1b	11					7				
Type 2	82					40				
Additional surgery	5 (1.6%)	12 (5.9%)	.26	.09 - .76	<.01	0	10 (6.2%)			<.01
30 days mortality	3 (1.0%)	2 (1.0%)			1.0	2 (1.2%)	1 (0.6%)			1.0

CI: confidence interval; EVAR: endovascular aneurysm repair; OAR: open aneurysm repair; PSM: propensity score matching

Supplementary Table 3 Cost comparison between EVAR and OAR in AAA ruptured cases

	EVAR	OAR	P-value
**Number of cases**	14	25	
**Patient background**			
Age (year)	81 (70-84)	75 (67-78)	.13
Male gender (%)	11 (79%)	25 (100%)	.04
AAA diameter (mm)	62 (54-76)	68 (60-84)	.10
Hb (g/dL)	10.5 (7.9-12.9)	11.3 (10.1-13.8)	.30
COPD (%)	3 (21%)	0 (0%)	.04
Atrial fibrillation (%)	0 (0%)	3 (12%)	.29
**Surgical outcomes**			
Operation time (min)	122 (92-147)	222 (173-265)	<.01
Bleeding (ml)	120 (44-268)	2403 (1635-3970)	<.01
ICU stay (day)	1.5 (1.0-10.5)	2.0 (1.0-9.3)	.49
Post operative length of hospital stay (day)	19.0 (6.8-52.0)	24.0 (13.5-48.0)	.50
30 days mortality (%)	2 (14%)	2 (8%)	.61
**Costs**			
DPC charges	772 (269-1151)<br>15%	1057 (650-1789)<br>41%	.29
Surgical procedure fees	773 (494-890)<br>15%	760 (543-877)<br>29%	.19
Surgical material costs	2213(189-2677)<br>44%	300 (264-450)<br>12%	<.01
Intravenous drug costs	38 (23-60)<br>1%	51 (38-69)<br>2%	.11
Anesthesia management fees	102 (82-107)<br>2%	146 (116-240)<br>6%	<.01
ICU management fees	134 (72-513)<br>3%	216 (120-866)<br>8%	.17
**Total hospitalization costs**	5038 (3970-6616)	2584 (2094-6385)	.17

Categorical variables are presented as number (%), Continuous variables are presented as medians (interquartile range), Costs are listed in Japanese yen $\times 10^3$.

AAA: abdominal aortic aneurysm; BMI: body mass index; COPD: chronic obstructive pulmonary disease; DPC: diagnosis procedure combination; EVAR: endovascular aneurysm repair; Hb: hemoglobin; ICU: intensive care unit; OAR: open aneurysm repair
